# Drug shortages in Israel, revisited: a bitter pill to swallow

**DOI:** 10.1186/s13584-024-00600-4

**Published:** 2024-03-18

**Authors:** Eyal Schwartzberg, Eli Marom, Alla Vishkautzan, Einat Gorelik, Segev Shani

**Affiliations:** 1 School of Pharmacy, Health Sciences Faculty, Ben Gurion University, Beer Sheva, Israel; 2 Pharmaceutical Society of Israel, Tel Aviv, Israel; 3 Pharmaceutical Division, Ministry of Health, Jerusalem, Israel; 4 Pharmacovigilance and Risk Management Department, Pharmaceutical Division, Ministry of Health, Jerusalem, Israel; 5 Department of Health Management and Policy, Guilford Glazer Faculty of Business and Management and Health Sciences Faculty, Ben Gurion University, Beer Sheva, Israel

**Keywords:** Drug shortages, Regulatory perspectives, Healthcare system, Patient access, Mitigation strategies

## Abstract

**Background:**

In 2017, we published an article addressing drug shortages (DS) in Israel, exploring regulatory perspectives, challenges, and potential solutions. Since then, DS remain a significant concern for patients, healthcare providers, and policymakers globally. In this updated article, we revisit the topic, providing new insights, data, and analysis on the current DS landscape in Israel, efforts to mitigate them, and propose strategies to combat this escalating issue.

**Methods:**

We conducted a comprehensive search of the Israeli Ministry of Health (MOH) DS database, spanning from 2014 to the present. We extracted DS numbers and their reasons. Further searches on the Israeli MOH website, pharmaceutical division archives, and the internet yielded official MOH publications and correspondence regarding regulatory responses to DS from 2017 onwards. Additionally, two specific cases of DS were examined to analyze their handling. Recent activities and publications from the Israeli MOH aimed at reducing DS were also reviewed.

**Results:**

Between 2014 and 2022, DS surged 2.66-fold. Total DS were 3228; 672 due to commercial reasons, and 2556 to operational reasons (20.5% and 79.5% respectively). The average duration of intermittent DS increased 1.56-fold, from 85 to 133 days. Manufacturers informed the MOH 22 days prior to actual shortage on average. Analyzing 2022's DS (640) by ATC groups, prominent categories included nervous system drugs (18%), drugs acting on the alimentary tract and metabolism (14%), and dermatologicals (11%). Operational DS in 2022 (n = 564) were primarily due to stock delivery delays (38%), stock over-utilization (12%), and raw material shortages (9%). Sixteen official MOH publications on DS were identified from 2017 onwards. Moreover, two high-impact DS case studies were examined.

**Conclusion:**

Despite routine monitoring by the Israeli MOH and updating the DS policy throughout this period, DS persist, intensifying annually and posing serious health risks. This trend mirrors international patterns, affecting countries globally. In Israel's uniquely structured healthcare system, with its swift stakeholder cooperation and implementation capabilities, more effective DS management is conceivable. We propose ten universally applicable rules to address DS challenges.

## Background

The issue of drug shortages is pervasive on a global scale, and Israel is no exception [1–7]. Patients' dependent on prescription medications may find themselves grappling with the unavailability of essential drugs, leading to treatment delays and potential adverse outcomes. While Israel boasts an advanced healthcare system, it, like many other nations, continues to grapple with the growing problem of drug shortages.

In our previous article published in 2017 [8], we underscored the emerging issue of drug shortages (DS) in Israel, a fact substantiated by the numerous instances observed between 2013 and 2017. As we delve into the current landscape, it becomes evident that the problem has escalated dramatically, evolving into a daily predicament affecting both patients and healthcare providers. Upon close examination of the present factors underlying these DS, we observe that they can be broadly categorized, as we expounded upon in our 2017 manuscript, into two principal causes: commercial reasons and operational reasons.

Within the framework of the Israeli Ministry of Health's (MOH) strategy to combat and address DS, Marketing Authorization Holders (MAHs) are required according to Regulation 7(6) of the Pharmacists Regulations—Medicinal Products (1986) to Establish regular and continuous supply of the product. Therefore, MAHs are bound by legal obligation to report all anticipated drug shortages to the pharmacovigilance department within the pharmaceutical division of the MOH. As per Israeli MOH Procedure #104: "Reporting on the intention to cease marketing or renew the registration of a medicinal product", these reports entail comprehensive details regarding the reasons for the shortage (commercial or operational) and the projected duration of the DS [9]. Additionally, if the ministry determines that a potential crisis is imminent due to issues affecting the quality, efficacy, and safety of a medicinal product, which could be exacerbated by DS, the ministry may choose to activate its procedure #84: "Transferring information and managing crises related to the medicinal products" [10]. However, it's important to note that this procedure primarily discusses general causes and responses that might lead to a crisis, without specifically addressing DS.

To acquire a more profound understanding of the aforementioned DS mechanics [11], we delve into the subsequent elaboration of commercial reasons for DS and operational reasons for DS as defined in Procedure #104 [9].

### Commercial reasons for DS

These could be defined as DS that occur when manufacturers decide to stop producing a drug due to low profitability. This can happen if a drug becomes generic or if the demand for the drug decreases. In some cases, manufacturers may also decide to focus on producing other more profitable drugs, which can result in shortages of less profitable drugs. Additionally, the manufacturer may decide that regulatory demands made by the applicable regulatory agency for a specific drug are too expensive to invest in, and thus withdraw the drug from the market.

### Operative reasons for DS

1. *Regulatory issues* Drug shortages can occur when manufacturers fail to comply with regulatory requirements, such as good manufacturing practices (GMP) or product labeling requirements. In some cases, regulatory authorities may also require the withdrawal of a drug from the market, which can lead to a shortage. Additionally, regulatory issues can delay the approval and launch of new drugs, while changes in existing regulations can also lead to shortages of certain drugs.
2. *Manufacturing problems* Manufacturing problems can occur due to equipment breakdowns, raw material shortages, or issues with production lines, which can lead to a decrease in the supply of drugs as well as other manufacturing issues.
3. *Supply chain disruptions and distribution problems* Distribution problems can occur due to issues with shipping, transportation, or storage, which can lead to damaged goods and delays in availability of drugs.
4. *Increased demand* Increased demand for a particular drug, such as during a disease outbreak or due to changes in prescribing patterns, can lead to shortages. Additionally, the shortage of one drug may lead to shortage of drug/s with the same activity or from the same class that may be used as alternatives.
5. *Natural disasters and wars* Natural disasters, such as hurricanes, earthquakes or pandemics which can result in a disruption of the workforce, disrupt drug manufacturing and distribution, leading to shortages. In some cases, natural disasters may also damage drug manufacturing facilities, making it difficult to produce drugs.

In this study, we explored the current extent of the drug shortage problem in Israel compared to previous years from 2014 to 2022, analyzed two case studies of DS occurring between 2018 and 2022 in Israel, examined their resolutions, discuss potential solutions to DS, and conclude by examining the implications of DS for the future of healthcare in Israel including potential recommendations for decision-makers (This article will not analyze COVID-19 specific shortages handling).

## Methods

To thoroughly explore the landscape of drug shortages in Israel and gain a comprehensive understanding, the following methodology was employed:

1. *Database analysis* The Israeli MOH maintains a comprehensive database that catalogues all recorded instances of drug shortages in Israel since 2014. This repository of information about DS of medicinal products is made readily accessible through the MOH website [12], providing a platform for engagement by diverse stakeholders including pharmaceutical companies, Health Maintenance Organizations (HMOs), and healthcare providers. Within this database, a trove of information is encapsulated, encompassing the details of the shortage including the expected duration, the root causes of DS and the measures taken to rectify them. It is important to note that as there is no available data regarding the total number of drugs approved in Israel, the results are presented in nominal values and cannot be normalized to the number of approved products.
2. *Literature review and regulatory landscape analysis* A meticulous review was undertaken to thoroughly explore the existing regulatory landscape and publications pertinent to drug shortages within the context of Israel. This process was augmented by an exhaustive search to extract insights into the designated guidelines introduced by the MOH after 2017. These guidelines are strategically designed to address and manage the multifaceted challenges engendered by drug shortages [13, 14]
3. *Case studies identification and analysis* Two distinct case studies, each emblematic of DS occurrences in Israel since 2017, were meticulously identified and subjected to comprehensive analysis. These were identified as significant since they were part of a global drug shortage and triggered proactive intervention by the Israeli MOH (as described in the Results section). The initial case study delves into the transient shortage of valsartan, which was precipitated by concerns over cancer risk due to impurities in 2018. The subsequent case study critically examines the scarcity of semaglutide (Ozempic) in 2022, a situation stemming from amplified demand due to off-label usage compounded by constrained stock availability, similar to the situation in the Europe and the United States [15, 16]. These case studies underwent meticulous scrutiny to unveil the intricacies of their unfolding, the approaches employed to address them, and the broader implications they hold.

By embracing these methodological frameworks, a comprehensive and holistic comprehension of the intricate dynamics and potential resolutions pertinent to the pressing challenge of drug shortages within the Israeli healthcare realm has been achieved.

## Results

**Fig. 1:**
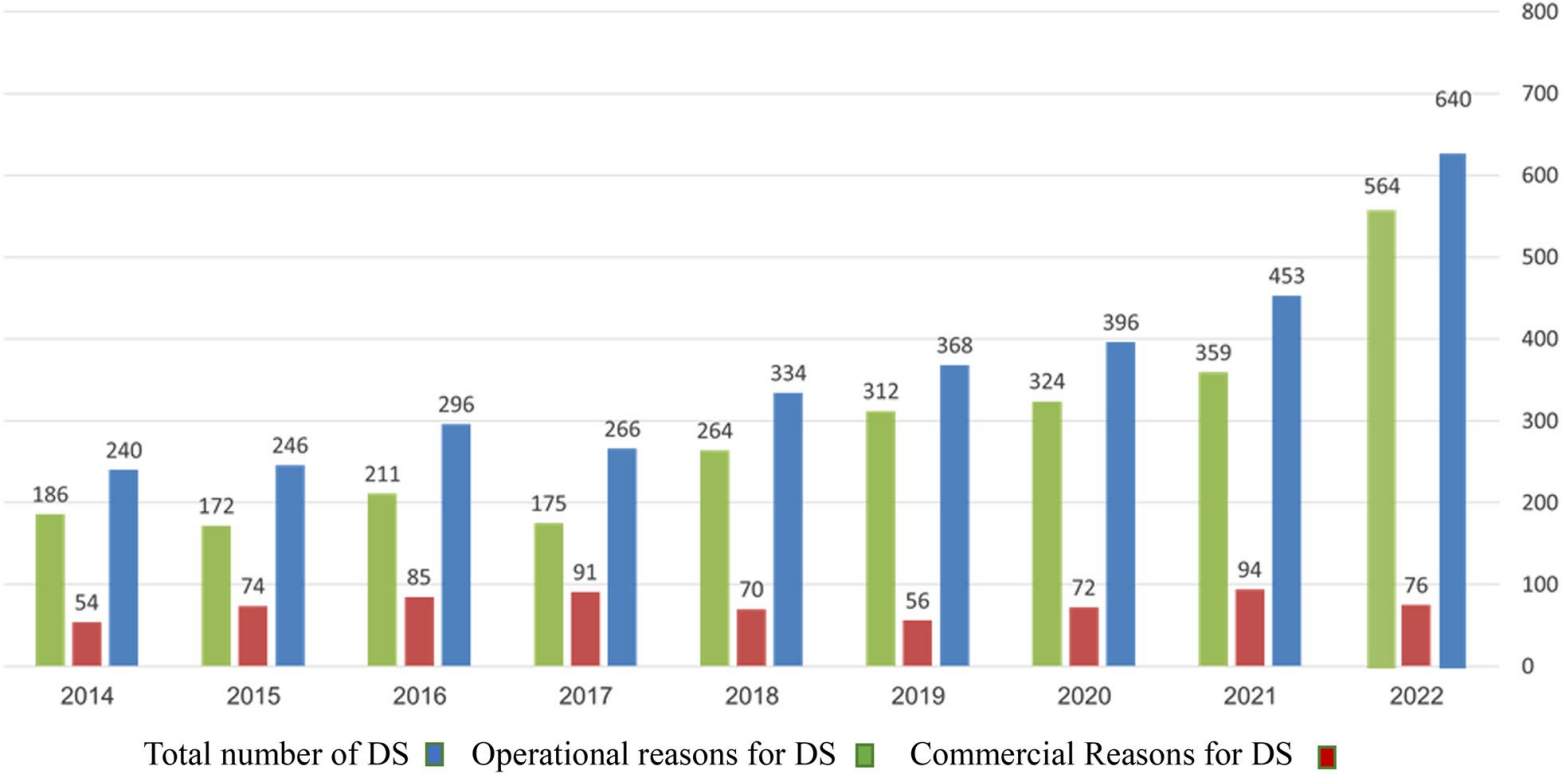
Reasons and numbers of DS in Israel between 2014 and 2022

Figure 1 displays the number of drug shortages reported in Israel and the reason for the shortage: commercial or operational.

Upon examination of the data presented in Fig. 1, readers can readily discern a notable surge, approximately 2.6-fold, in the number of DS between 2014 and 2022. Over the span of this period, the cumulative count of DS reached 3268 instances, with 671 occurrences attributed to commercial factors and 2597 occurrences rooted in operational considerations (accounting for 20.5% and 79.5% respectively).

**Fig. 2:**
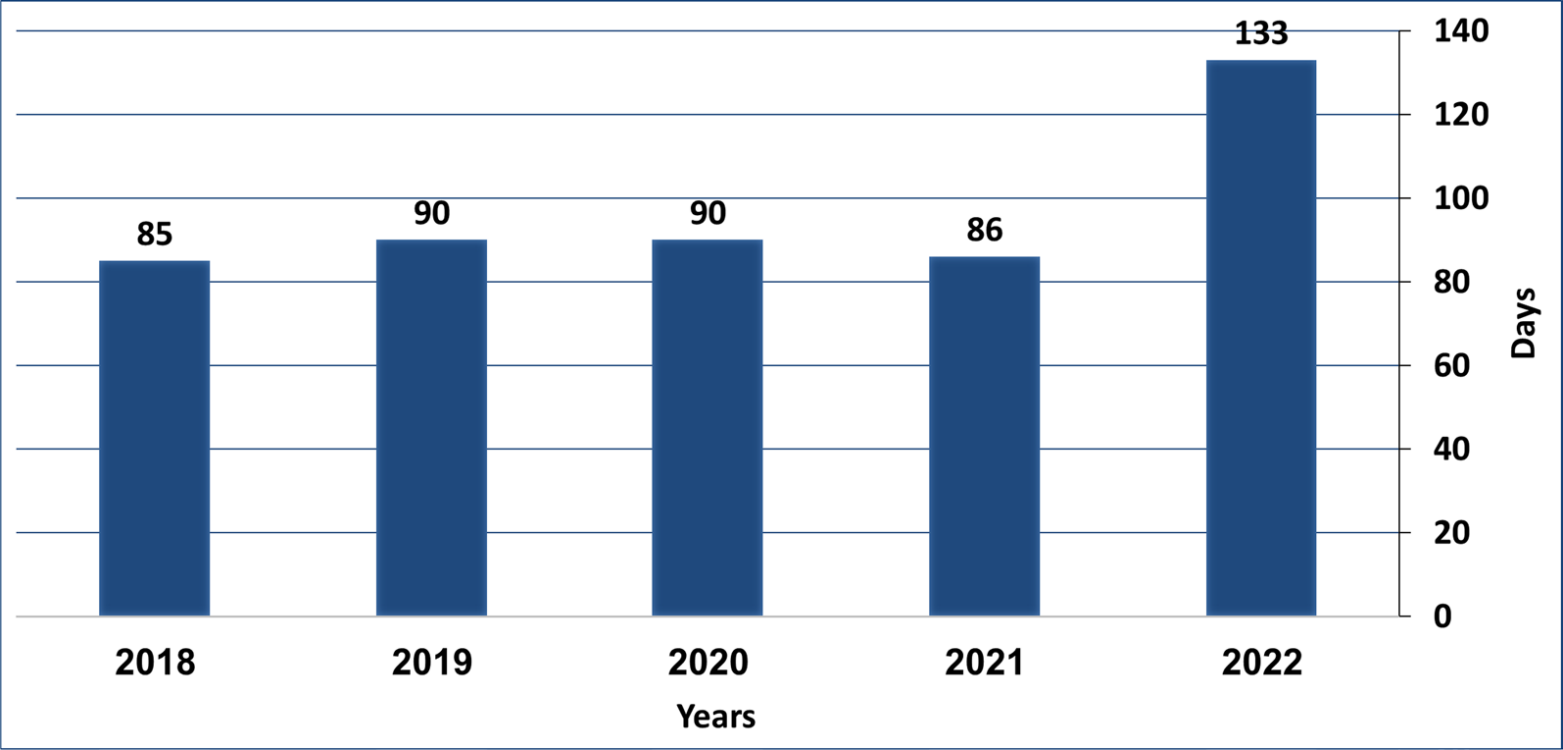
Shortage duration (average number of days) during 2018–2022

Furthermore, a compelling trend is unveiled in Fig. 2, depicting a 1.56-fold augmentation in the average duration of intermittent DS, evolving from 85 to 90 days between 2018 and 2021 to 133 days in 2022. Of noteworthy significance, the average interval of time between the decision to discontinue and notification of the Israeli MOH by the MAHs was recorded as 22 days which is a short period for handling the DS. In 2021, 70% of the DS notifications were provided in less than 30 days prior to the actual shortage, while in 2022, 88% of the DS notifications were provided in less than 30 days prior to the actual shortage.

This analysis underscores the substantial shifts in the landscape of DS, revealing patterns that illuminate the challenges faced and developments observed in the realm of pharmaceutical supply and availability.

For a comprehensive overview, Table 1 elucidates the Anatomical Therapeutic Chemical (ATC) categories, quantities, and percentages of medicines affected by DS based on the ATC classification, observed in both 2022 and 2013–2015. This analysis sheds light on the evolving trends in drug availability and the shifting prevalence of DS within distinct therapeutic classes.

**Table 1:** Type, number, and percentage of DS per year by ATC category, 2022 versus 2013–2015

Years	2022		2013–2015	
ATC category	DS per year	%	DS per year (average)	%
Alimentary tract and metabolism	91	14	15	7
Blood and blood forming organ	26	4	9	4
Cardiovascular system	56	9	33	15
Dermatologicals	70	11	13	6
Genito-urinary system and sex hormones	33	5	17	7
Systemic hormonal preparations, excluding sex hormones and insulins	11	2	4	2
Anti-infectives for systemic use	67	10	36	16
Antineoplastic and immunomodulating agents	64	10	13	6
Musculo-skeletal system	23	4	13	6
Nervous system	113	18	47	21
Antiparasitic products, insecticides, and repellents	6	1	1	0
Respiratory system	50	8	8	3
Sensory organs	19	3	7	3
Various	11	2	9	4
Total	640	100	226	100

In 2022, drugs acting on the nervous system constituted 18% of the total, followed by drugs targeting the alimentary tract and metabolism at 14%, and dermatologicals at 11%. Subsequently, anti-infective drugs for systemic use and antineoplastic and immunomodulating agents accounted for 10% each. Cardiovascular drugs and respiratory nervous drugs stood at 9% and 8% respectively. Akin findings with minor fluctuations were observed in the European Commission's assessment in 2021 [5].

Additionally, when scrutinizing the most recurrent DS incidents of medicines as per their ATC groupings spanning the period 2013–2015, the subsequent trends came to the fore: nervous system drugs comprised 21%, followed by anti-infective drugs for systemic use at 16%, cardiovascular drugs at 15%, and drugs for the alimentary tract and metabolism, along with those acting on the genito-urinary system and sex hormones, both at 7%. However, there is a significant difference when comparing the total number of DS per year in 2022 (640) with that observed between 2013 and 2015 (226).

**Fig. 3:**
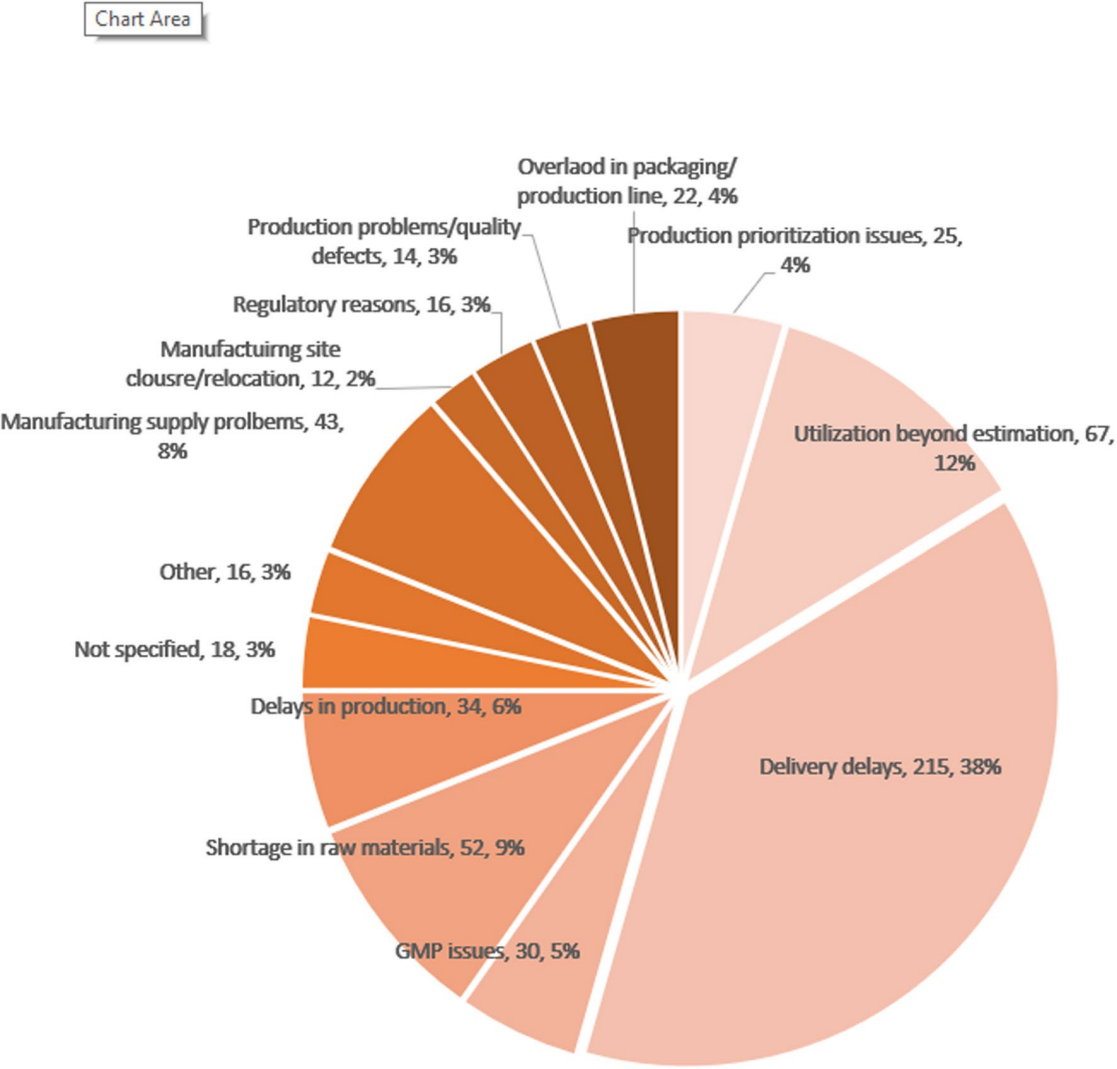
Drug shortages due to operational reasons in 2022 (n = 564)

Figure 3 provides a detailed breakdown of the underlying causes of drug shortages attributed to commercial reasons in the year 2022. The findings distinctly highlight the predominant factors contributing to DS, with the following five primary causes identified: Delay in Drug Delivery/Supply (38%), Over-Utilization of Medicines in Israel (12%), Raw Materials Shortage (9%), Manufacturing Supply Problems (8%), Delays in Production (6%).

Collectively, these factors constitute 70% of the total DS instances attributed to operational reasons, and they also account for 64% of the overall DS incidents observed in the year 2022.

### MOH publications on DS, 2017–2022

Upon reviewing official publications related to DS, we identified four key findings. The process involved a comprehensive search conducted in both Hebrew and English on the MOH website and the Pharmaceutical Division archives. The search employed various combinations of keywords, including "shortage," "drug(s)," and "discontinuation(s)."

The outcomes of this search yielded a total of 17 results, categorized as follows:

1. Seven spokesperson official announcements concerning valsartan [17–19], tocilizumab (Acterma) [20], Losartan [21, 22] and Champix (Varenicline) [23].
2. Four procedures addressing different aspects of drug shortages [2, 13, 24, 25].
3. One aggregated list detailing the discontinuations of various drugs [26].
4. Four instances of official correspondence regarding semaglutide [15, 27, 28] and valproic acid shortage [29].

These findings exemplify the outcomes of our meticulous search process, highlighting the range of official documents retrieved from the MOH and Pharmaceutical Division resources.

### Case studies

#### Valsartan—detection of NDMA substance in Mylan's Valsartan leading to medication withdrawal

In response to the European Medicines Agency's (EMA) alert regarding the presence of carcinogenic materials, specifically N-nitrosodimethylamine (NDMA), during the manufacturing of the active ingredient Valsartan, used for treating hypertension and heart failure, the Israeli MOH issued a series of public announcements and advisories from July to December 2018. These communications aimed to address emergent concerns.

During this period, the MOH published four separate notices directed at the general public and healthcare practitioners. Recognizing the potential risk, the MOH decided to initiate a recall of all batches of Mylan's Valsartan from pharmacies. It is noteworthy that the utilization of the MOH Procedure #84 titled "Pharmaceutical Crisis Management" was invoked in this instance, according to which, a broad multidisciplinary team was convened to manage the upcoming shortage. Moreover, the issue was discussed with medical experts in the field of hypertension and the four Sick Funds (HMOs) were informed to coordinate the transfer of patients to alternative treatments.

The MOH provided specific instructions regarding the shortage situation, which included:

- Attending physicians were entrusted with determining the appropriate course of treatment based on individual clinical circumstances and the guidelines of the Sick Funds (HMOs).
- The MOH emphasized to the public that discontinuing the use of the remaining medication was not advised, as halting treatment could disrupt blood pressure equilibrium and potentially harm patients. Continuing medication usage was considered safer than discontinuing it.
- Medications containing VALSARTAN, not manufactured by Mylan, were not deemed in need of replacement.
- The MOH committed to ongoing monitoring of the situation, pledging to update the public with any new information received.

Approximately 2 months later, the shortage was resolved, and a regular supply of the drug was restored.

#### Ozempic (semaglutide)—drug shortages due to off-label use for obesity treatment

Semaglutide, marketed as Ozempic, is a Glucagon-like peptide-1 (GLP-1) analogue indicated for treating type II diabetes via weekly subcutaneous administration. Following successful clinical trials, Novo Nordisk, the pharmaceutical company owning the product, sought additional indications, including obesity treatment for individuals at risk or suffering from overweight, including childhood obesity. This prompted the regulatory approval of semaglutide under the trade name Wegovy for this new use. As this expansion unfolded, clinicians began prescribing Ozempic as an off-label treatment for obesity, especially considering Wegovy's limited availability globally and its delayed approval in Israel which occurred only in 2022 [15, 16].

With a sudden upsurge in demand due to off-label use, availability issues ensued due to restricted national stockpiles and the Marketing Authorization Holder's limited capacity to cater to this unforeseen demand. Such shortages occurred both in the United States and Europe [15, 16]. Consequently, Novo Nordisk declared a drug shortage, impacting both diabetic and obesity patients. This led to periodic but significant gaps in drug availability. To counter this crisis, the MAH intensified semaglutide import efforts to Israel and cooperated with the MOH in managing the situation.

In response, the Israeli MOH issued guidelines and clinical instructions to prescribers, advocating rational semaglutide utilization emphasizing prioritized use for diabetic patients. The MOH also released directives to community pharmacists, underscoring the attending doctor's role in ensuring appropriate treatment and continuity of care. While acknowledging the validity of off-label use, these instructions stressed pharmacists' inability to prioritize patients based on stock levels. In cases of shortage, pharmacists were directed to refer patients back to their doctors for suitable alternatives.

As the situation persisted, the National Diabetes Council recommended to the MOH that oral semaglutide (trade name Rybelsus) could serve as an alternative to injectable semaglutide to address treatment gaps. Eventually, the MAH managed to meet the demand, although the potential for future shortages remains due to potential surges in demand. In this instance, the MOH chose to disseminate instructions solely to healthcare professionals, after consulting with experts in the field of diabetes and obesity, without a broader public outreach approach.

## Discussion

The results presented in this manuscript study illuminate the trends and factors associated with DS in Israel between 2014 and 2022. This comprehensive analysis provides valuable insights into the multifaceted dynamics driving the occurrence of drug shortages. The findings reveal several noteworthy patterns within the pharmaceutical landscape. One of the most striking observations is the significant increase in DS, with a 2.66-fold rise observed over the study period. This rise is of particular concern due to its potential impact on patient access to essential medications. Throughout this timeframe, a total of 3268 DS incidents were recorded, with commercial reasons accounting for 20.5% and operational reasons for the remaining 79.5% of these DS. The significant difference between commercial and operational reasons may be explained in the fact that the aim of pharmaceutical companies is to maximize revenues and hence their aim is to sell their products. Therefore, unless local or global market conditions change, once a pharmaceutical company decides to launch a product in a specific territory, there is no logic in discontinuing sales. Therefore, most shortages are operational in their nature due to supply chain or manufacturing issues. Nevertheless, we should point out that whether the DS is caused by commercial or operational reason is based on the companies' self-reports which might be biased or subject to individual interpretation.

A closer examination of the average duration of intermittent DS unveils a 1.56-fold increase from 85 to 90 days between 2018 and 2021 to 133 days in 2022. Moreover, the short time between reporting the DS and its beginning—22 days in 2022, does not provide the healthcare system with sufficient time to adequately prepare alternative treatments. This extended disruption in medicine availability raises concerns about potential consequences for patient outcomes, underscoring the importance of comprehending the underlying reasons behind these interruptions. Moreover, the study delves into the categorization of discontinued medicines based on their Anatomical Therapeutic Chemical (ATC) classification. Notably, nervous system drugs consistently ranked high both in 2022 and the earlier period of 2013–2015, highlighting the vulnerability of this therapeutic category to DS. These trends resonate with observations from the European Commission, implying that the challenges faced are not exclusive to Israel but might reflect broader issues within the pharmaceutical industry [1].

The MOH encounters some limitations when addressing the DS challenge: the lack of specific legislation concerning DS including enforcement measures. Procedure 84, which primarily focuses on crisis management related to medicinal products with new information that could seriously impact public health, is not specifically designed to address DS as a crisis scenario. Additionally, within the pharmacy ordinance, its regulations, and subsequently in procedure 104 (reporting on the intention to cease marketing or renew the registration of a medicinal product), there are no penalties established for companies that fail to report DS incidents on time or maintain minimum stock levels.

In light of these challenges, a comprehensive approach is imperative to effectively manage and mitigate the impact of DS. The complexity of the issue spans low-, middle-, and high-income countries, making it universal and pertinent to OECD countries, and addressed by relevant leading regulatory agencies such as the US food and drug administration (FDA), US homeland security and governmental affairs and the EMA [1–7]. Israel's unique healthcare ecosystem and the regulatory challenges faced by the MOH necessitate a multi-faceted strategy towards mitigating DS.

Recommendations:

Although this manuscript describes issues related to drug shortages in Israel, most findings are common with other countries and healthcare systems. Hence, part or most of the recommended policies and practices that can be implemented in Israel, can also be implemented and are likely to be applicable in other countries:

1. *Defining critical drug shortages* Recognizing the absence of a clear definition of "critical" drug shortages within Israeli legislation, there is an urgent need to establish a comprehensive framework that identifies drugs where shortages can lead to severe health consequences, such as narrow therapeutic index drugs. This framework should meticulously outline specific criteria for assessing the level of risk associated with a shortage, thus enabling timely and effective interventions, such as the availability of generic or therapeutic alternatives. A collaborative effort involving healthcare professionals, regulators, and industry stakeholders can ensure that these criteria are evidence-based and adaptable to various healthcare scenarios.
2. *Revisiting procedure 84/implementing specific DS processes* The current version of Procedure 84 predominantly addresses crisis management for faulty medicinal products. However, it is imperative that the competent regulatory authority should broaden its scope to encompass drug shortages as a crisis scenario. This extension should include well-defined guidelines for rapid response and the necessary actions to be taken in the event of a drug shortage by the MoH after consulting with the relevant medical organization in the therapeutic area. Such proactive measures will ensure that relevant stakeholders are informed promptly and are able to coordinate efforts effectively, thereby minimizing the impact on patient care.
3. *Strengthening reporting and penalties* To reinforce the regulatory framework, revisions to the pharmacy ordinance and Procedure 104 should incorporate enforcement possibilities for companies failing to report drug shortages on time or maintain minimum stock levels. By imposing significant consequences for non-compliance, a stronger incentive will be created for pharmaceutical companies to adhere rigorously to regulatory requirements, promoting a more robust and accountable pharmaceutical landscape.
4. *Facilitate rapid importation of drugs in shortage* Leveraging Israel's distinct healthcare landscape, a collaborative approach involving the four Sick Funds (HMOs) under the national health insurance law can expedite the importation of essential medications facing shortages. A dedicated committee comprising of representatives from the MOH, HMOs, pharmaceutical manufacturers, and regulatory bodies should be established. This committee's combined expertise will facilitate swift decision-making, ensuring the efficient procurement of critical medications from global stockpiles, thereby maintaining uninterrupted patient access during periods of scarcity.
5. *Stockpiling strategy* A comprehensive stockpiling and Special Long-Term Expiry Programs (SLEP) initiative can be expanded upon, guided by an analysis of primary reasons for supply chain disruptions. This strategy will involve identifying crucial medicines susceptible to shortages and implementing a contingency plan that supports stockpiling. The SLEP approach should be extended to certain critical medications, enhancing the maintenance of adequate stock levels during challenging times.
6. *Collaborative crisis committee* Establishing a dedicated committee comprising representatives from the MOH, healthcare professionals, the pharmaceutical industry, and patient advocacy groups is pivotal for effective crisis response. Empowered to provide clear and consistent instructions to the public, healthcare practitioners, and other stakeholders during drug shortage scenarios, this committee will ensure a coordinated and unified approach to tackling challenges.
7. *Enhanced communication* The MOH should prioritize open dialogues with MAHs and healthcare professionals, fostering proactive communication to anticipate potential disruptions in the supply chain. To address public concerns, an effective communication system must be implemented, including a dedicated "hot line" information center operated by the MOH. Leveraging public media platforms will amplify communication efforts, disseminating accurate information to the wider public and building transparency, trust, and informed decision-making.
8. *Cross-functional training* Developing targeted training programs for healthcare professionals in managing drug shortages is crucial. These programs should encompass alternate treatment options, rational drug utilization, and effective patient communication. Allowing pharmacists to substitute drugs facing shortages with agreed-upon alternatives within the same ATC classification can ensure informed decision-making during shortages.
9. *Adaptive regulatory framework* In tandem with a revised definition of critical drug shortages, the regulatory framework must remain adaptable to emerging challenges. Regular review and updates will ensure its continued effectiveness in addressing changing circumstances, promoting agility and resilience within the healthcare system including risk–benefit evaluation of approving the use of non-GMP compliant products in case of shortages.
10. *Incentives for manufacturers* Encouraging pharmaceutical manufacturers to produce essential medications with limited market demand is essential for maintaining a stable drug supply. The Israeli healthcare system should consider introducing incentives, such as financial support, grants, tax benefits, or subsidies, for manufacturers committing to producing vital drugs that may have limited commercial appeal. Extending patents or market exclusivity for drugs facing shortages can further incentivize sustained production, safeguarding a consistent supply of medications critical to public health.

Recently, The EMA Medicine Shortages Steering Group published recommendations on tackling shortages of medicinal products which are very similar to the above recommendation emphasizing implementation of regulatory flexibilities [30]. Adopting these recommendations will empower Israel to build a resilient approach to managing DS, aligning with the unique features of its healthcare structure. By integrating regulatory reforms, clear guidelines, collaborative efforts, and proactive communication, the impact of DS on patient care can be minimized, while the overall preparedness of the healthcare system to handle such crises effectively can be enhanced.

## Conclusion

This study unequivocally underscores the ongoing need for vigilant monitoring of drug shortages and the implementation of strategies to mitigate their impact on patient care and safety. It emphasizes the importance of continued collaboration among stakeholders within the healthcare ecosystem to ensure unfettered access to necessary medications and the promotion of patient safety. Such collaborative efforts are indispensable in navigating the complex landscape of DS, and they underscore the healthcare community's dedication to upholding patient welfare in the face of these challenges.

## Data Availability

The datasets used and/or analysed during the current study are available from the corresponding author on reasonable request.

## Abbreviations

ATC: Anatomical Therapeutic Chemical
DS: Drug shortage
EMA: European Medicine Agency
FDA: Food and drug administration
GMP: Good Manufacturing Process
HMOs: Health Maintenance Organizations
MAHs: Marketing Authorization Holders
MOH: Ministry of Health
OECD: Organization for Economic Co-operation and Development
SLEP: Special Long-Term Expiry Programs
